# Association Between US Physician Malpractice Claims Rates and Hospital Admission Rates Among Patients With Lower-Risk Syncope

**DOI:** 10.1001/jamanetworkopen.2020.25860

**Published:** 2020-12-15

**Authors:** James Quinn, Sukyung Chung, Audrey Murchland, Giovanni Casazza, Giorgio Costantino, Monica Solbiati, Rafaello Furlan

**Affiliations:** 1 Department of Emergency Medicine, Stanford University, Stanford, California; 2 Stanford University School of Medicine, Stanford, California; 3 Dipartimento di Scienze Biomedichee Cliniche “L. Sacco,” Universita' degli Studi di Milano, Milano, Italy; 4 Fondazione IRCCS Ca' Granda, Ospedale Maggiore Policlinico, Milano, Italy; 5 Department of Internal Medicine, Humanitas University, Rozzano, Italy

## Abstract

**Importance:**

The US Government Accountability Office has changed its estimate of the annual costs of defensive medicine, largely because it has been difficult to objectively measure its impact. Evaluating the association of malpractice claims rates with hospital admission rates and the costs of admitting patients with low-risk conditions would help to document the impact of defensive medicine. Although syncope is a concerning symptom, most patients with syncope have a low risk of adverse outcomes. However, many low-risk patients are still admitted to the hospital, with associated costs of more than $2.5 billion per year in the US.

**Objective:**

To assess whether hospital admission rates after emergency department visits among patients with lower-risk syncope are associated with state-level variations in malpractice claims rates.

**Design, Setting, and Participants:**

This cross-sectional study of emergency department visits among patients with lower-risk syncope used deidentified data from the Clinformatics Data Mart database (Optum). Lower-risk syncope visits were defined as those with a primary diagnosis of syncope and collapse based on *International Classification of Diseases, Ninth Revision, Clinical Modification* code 780.2 or *International Classification of Diseases, Tenth Revision, Clinical Modification* code R55 that did not include another major diagnostic code for a condition requiring hospital admission (such as heart disease, cancer, or medical shock) or an inpatient hospital stay of more than 3 days. These data were linked to publicly available data from the National Practitioner Data Bank pertaining to physician malpractice claims between January 1, 2008, and December 31, 2017. The 2 data sets were linked at the state-year level. Data were analyzed from October 2, 2019, to September 12, 2020.

**Main Outcomes and Measures:**

The association between the rate of hospital admission after emergency department visits among patients with lower-risk syncope and the rate of physician malpractice claims was assessed at the state-year level using a state-level fixed-effects model. Standardized costs obtained from the Clinformatics Data Mart database were adjusted for inflation and expressed in 2017 US dollars using the Consumer Price Index.

**Results:**

Among 40 482 813 emergency department visits between 2008 and 2017, 519 724 visits (1.3%) were associated with syncope. Of those, 234 750 visits (45.2%) met the criteria for lower-risk syncope. The mean (SD) age of patients in the lower-risk cohort was 71.8 (13.5) years; 141 050 patients (60.1%) were female, and 44 115 patients (18.8%) were admitted to the hospital, representing an extra cost of $6542 per admission. The mean rate of physician malpractice claims varied from 0.27 claims per 100 000 people to 8.63 claims per 100 000 people across states and across years within states. A state-level fixed-effects regression model indicated that, for every 1 in 100 000–person increase in the physician malpractice claims rate, there was an absolute increase of 6.70% (95% CI, 4.65%-8.75%) or a relative increase of 35.6% in the hospital admission rate, which represented an additional $102 million in costs associated with this lower-risk cohort.

**Conclusions and Relevance:**

In this study, increases in physician malpractice claims rates were associated with increases in hospital admission rates and substantial health care costs for patients with lower-risk syncope, and these increases are likely associated with the practice of defensive medicine.

## Key Points

**Question:**

Are US physician malpractice claims rates associated with hospital admission rates after emergency department visits among patients with lower-risk syncope?

**Findings:**

In this cross-sectional study of 519 724 emergency department visits for syncope between 2008 and 2017, 19% of patients with lower-risk syncope were admitted to the hospital. A 7% absolute increase in the admission rate was found for every 1 in 100 000–person increase in the malpractice claims rate.

**Meaning:**

The study’s findings indicated that most lower-risk patients with syncope were not admitted to the hospital; however, increases in physician malpractice claims rates were associated with substantial increases in hospital admission rates and admission-associated costs among patients with lower-risk syncope.

## Teaser

This cross-sectional study assesses the association between physician malpractice claims rates and hospital admission rates after emergency department visits among patients with lower-risk syncope in the US.

## Introduction

It has been estimated that 3% to 4% of health care costs are associated with the practice of defensive medicine.^1^ In 2009, the US Government Accountability Office reported that these associated health care costs could be substantially reduced through tort reform to decrease the rate of malpractice claims.^2^ Since 2009, conflicting evidence on the consequences of tort reform for defensive medicine (defined as the practice of ordering medical tests, procedures, or admissions and consultations of doubtful clinical value to protect the prescribing physician from malpractice claims) has produced lower estimates of the costs associated with defensive medicine.^3^ However, these estimates still represent annual costs of $55 billion in the US.^4^

Studies have suggested that liability concerns are associated with physicians’ decision-making when physicians are asked to justify their decisions^5,6,7^ and that physician practice and usage patterns vary based on liability concerns.^8^ Data regarding the impact of those liability concerns on health care use and costs are mixed, and the estimated extent and direction of the impact vary depending on how the liability risk climate is measured.^9^ Most studies have used state tort reform as a proxy for liability, with inconsistent results. The use of radiographic testing was reported to vary among states with and without tort reform; however, other studies did not find a difference in Medicare costs among states with or without tort reform.^10,11,12,13^ The actual malpractice claims rate may be a better measure of the liability risk climate and physicians’ perceptions of risk. That is, physicians practicing in states with higher rates of malpractice claims and greater risk of malpractice litigation may be more risk averse and more likely to practice defensive medicine.^14^ The practice of defensive medicine can be detected in the use of excessive and/or unnecessary health care resources for patients with low-risk conditions.

Syncope is a highly noticeable but low-risk symptom that accounts for 1% to 2% of all emergency department (ED) visits.^15,16,17^ Most factors underlying the occurrence of syncope are benign, although syncope is occasionally (ie, in 1%-2% of cases) associated with substantial morbidity and, rarely, with mortality.^18,19^ Syncope is accordingly considered a low-risk symptom that can, in some cases, indicate a serious underlying condition; many low-risk patients with syncope are therefore admitted to the hospital, even in cases for which the value of hospitalization is questionable and generally not recommended.^4,5,6^ Despite these recommendations, there is substantial variability in admission practices. The US has the highest hospitalization rates and spends over $2.5 billion annually on hospitalization alone.^20^ In this study, we investigated defensive medicine and health care costs by assessing the association between the liability risk climate (measured by state-level malpractice claims rates) and hospital admission rates among patients with lower-risk syncope in the US.

## Methods

This study followed the Strengthening the Reporting of Observational Studies in Epidemiology (STROBE) reporting guideline for cross-sectional studies. The study was approved by the institutional review board of Stanford University and deemed exempt from informed consent because the data sets contained no identifiable participant information.

Data were obtained from the Clinformatics Data Mart database (Optum), which includes deidentified data from members of a large national insurance provider. From January 1, 2008, to December 31, 2017, the database contained approximately 16.5 million members per year, of whom 18% were Medicare beneficiaries. Approximately 90% of members in the data set were continually insured for 6 months, and members included in the database have been reported to compare favorably with most reference standards for the US population.^21^ The database contains claims data, including ED visits, hospital admissions, and procedural claims, for all members; for each claim, the database provides standardized costs for the data set, which reflect average payments made by insurance providers.

### Cohort Definition

**Figure 1.**
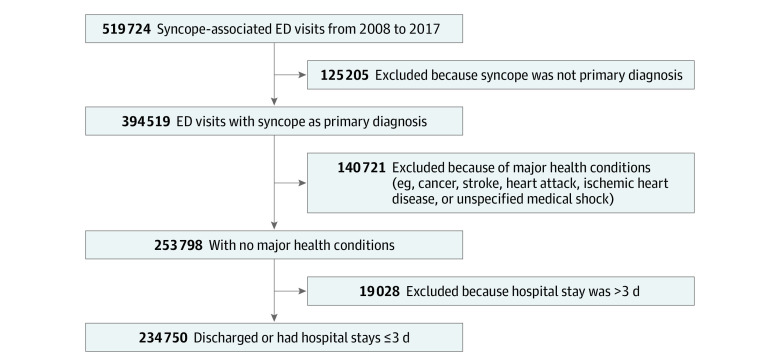
Development of Lower-Risk Syncope Cohort Participants were obtained using the Clinformatics Data Mart database (Optum). ED indicates emergency department.

To examine the malpractice environment associated with physicians’ judgment and hospital admission decisions, we created a cohort of patients with lower-risk syncope. These patients were more likely to be discharged after an ED visit during the study period (Figure 1). To identify patients for the cohort, we searched for those who had an ED visit for syncope that was associated with a primary diagnosis of syncope and collapse based on *International Classification of Diseases, Ninth Revision, Clinical Modification* (*ICD-9-CM*) code 780.2 or *International Classification of Diseases, Tenth Revision, Clinical Modification* (*ICD-10-CM*) code R55. These codes are most commonly used for syncope without clear underlying factors and have been reported to be 65% sensitive and 99% specific for syncope visits to EDs.^22^ We further refined the cohort into a lower-risk syncope group by excluding patients who presented to the ED with another major health condition that likely required hospital admission (eg, heart disease, stroke, cancer diagnosis, or medical shock) and patients for whom the ED visit resulted in a hospital stay of more than 3 days, which would have been indicative of a more serious condition.

Using deidentified data obtained from the Clinformatics Data Mart database, we defined a hospital admission as an ED visit associated with a hospital stay, an observational stay (based on *Current Procedural Terminology* codes 99218-20 and 99224-6), or inpatient charges of more than $1000 on day 0, 1, or 2 after the initial ED visit insurance claim. For each visit, we computed standardized costs associated with the ED visit and the subsequent inpatient stay. All costs were adjusted for inflation and expressed in 2017 US dollars using the Consumer Price Index.^23^ Data obtained from the Clinformatics Data Mart database were accessed through the Stanford Center for Population Health Sciences Data Core.

We then used publicly available state-level data from the National Practitioner Data Bank^24^ to assign malpractice claims rates associated with the ED visit. The database contains annual state-level data for all adverse actions, including malpractice claims, payments, and adverse-action reporting against all health care practitioners. When assessing the liability risk climate for physicians, we only considered malpractice claims against physicians with payments. To determine the rates per 100 000 people in each state per year, we used population estimates from the US Census Bureau, which are derived from decennial census data combined with estimated births, deaths, and migrations each year.^25,26^ The state-year level malpractice claims rate was then assigned to each ED visit based on the location (ie, state) in which the physician decided to admit or discharge the patient.

The purpose of the study was to assess the association between the malpractice liability environment and physician decision-making. The specific goal was to assess the decision to admit patients with lower-risk syncope, a condition for which most patients are discharged from the ED and physician judgment is involved. One would not, conversely, expect the malpractice environment to be associated with a diagnosis for which the decision to admit is unambiguous and not impacted by physician judgment. Acute appendicitis, for example, is a surgical diagnosis based on definitive findings. As a negative control for our model, we also examined the association between state variations in the malpractice claims rate and hospital admissions for appendicitis (using *ICD-9-CM* code 540.0 and *ICD-10-CM* code K35.2) using the same state-level fixed-effects model for the same population and period that were used for our lower-risk syncope cohort.

### Statistical Analysis

We compared age, sex, malpractice claims rate, and health care costs between patients who were admitted to the hospital vs discharged from the ED, using *t* tests for continuous variables and χ^2^ tests for dichotomous variables. We then assessed annual patterns in malpractice claims rates and syncope admission rates over the study period using fractional polynomial regression analysis (which uses flexible approaches to determine the functional form rather than a predefined linear or quadratic form to best characterize the association).^27^

A state-level fixed-effects linear regression model was used to estimate the association of the malpractice claims rate with the hospital admission rate. Because the state-level fixed-effects model used only within-state variation over time in the estimations, any time-invariant state-level confounders that might have had consequences for both the admission and malpractice claims rates (eg, unmeasured socioeconomic factors and physicians’ practice styles) were eliminated from the model. Data on demographic characteristics, age, and sex aggregated at the state-year level were controlled for as covariates. In all state-year–level analyses, frequency weights that reflected the total number of eligible ED visits per state-year in the data were applied. The Stata statistical package, version 16.0, was used for analysis. Data were analyzed from October 2, 2019, to September 12, 2020.

## Results

During the 10-year study period (2008-2017), there were 40 482 813 ED visits, with 519 724 visits (1.3%) associated with syncope (Table 1). Of those, 234 750 visits (45.2%) met the criteria for lower-risk syncope. The mean (SD) age of patients in the lower-risk syncope cohort was 71.8 (13.5) years; 141 050 patients (60.1%) were female, and 44 115 patients (18.8%) were admitted to the hospital. Compared with patients discharged from the ED, those admitted to the hospital were older (mean [SD] age, 71.2 [14.0] years vs 74.3 [10.7] years, respectively), less likely to be female (115 132 women [60.4%] vs 25 918 women [58.8%]), had a higher malpractice claims rate (mean [SD] rate, 2.49 [1.44] claims per 100 000 people vs 2.80 [1.57] claims per 100 000 people), and had ED visits that incurred $6542 higher hospital costs (mean [SD] cost, $4360 [$6533] vs $10 902 [$19 899]).

**Table 1.** Characteristics of Lower-Risk Syncope Cohort^**a**^

Characteristic	No. (%)		
	Total	Discharged from ED	Admitted to hospital
Patients, No.	234 750	190 635	44 115
Female sex	141 050 (60.1)	115 132 (60.4)	25 918 (58.8)
Age, y			
Mean (SD)	71.8 (13.5)	71.2 (14.0)	74.3 (10.7)
Category			
18-64	46 530 (19.8)	40 220 (21.1)	6310 (14.3)
65-75	79 511 (33.9)	64 994 (34.1)	14 517 (32.9)
76-97	108 709 (46.3)	85 421 (44.8)	23 288 (52.8)
Hospital costs, mean (SD), $			
Overall	5589 (10 752)	4360 (6533)	10 902 (19 899)
ED	3930 (6490)	4360 (6533)	2074 (5959)
Inpatient stay	1659 (9090)	NA	8828 (19 401)
Malpractice claims rate, mean (SD)^b^	2.55 (1.47)	2.49 (1.44)	2.80 (1.57)

Abbreviations: ED, emergency department; NA, not applicable.

^a^ The unit of observation was ED visit. Mean (SD) was used for continuous variables and No. (%) for categorical variables. For all variables, the difference between discharged and admitted groups was statistically significant (*P* < .001).

^b^ The malpractice claims rate ranged from 0.27 claims per 100 000 people to 8.63 claims per 100 000 people across state-year. A total of 233 099 ED visits were included; 1651 ED visits (0.7%) were excluded because they were missing state information.

**Figure 2.**
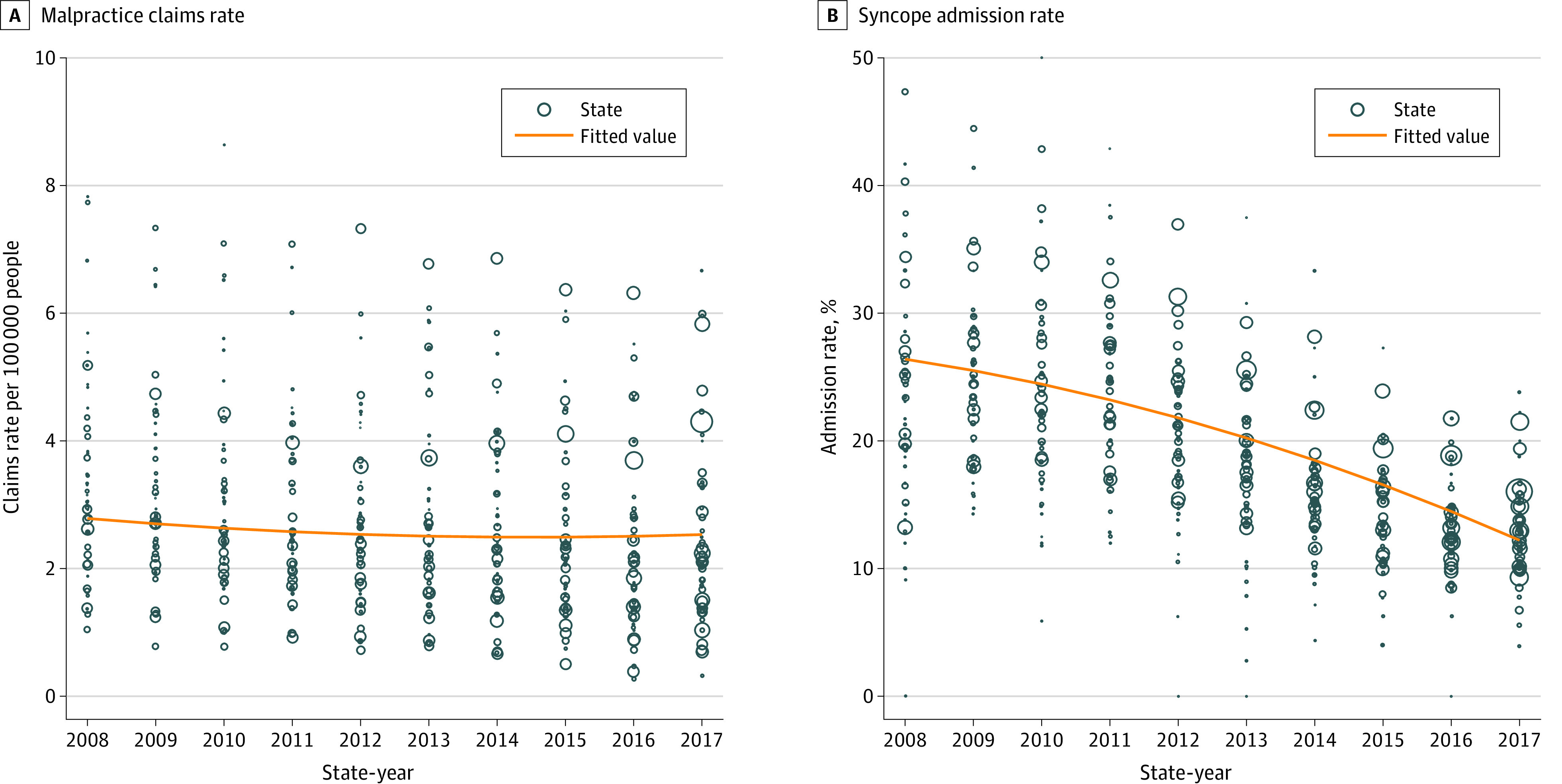
Variation of Malpractice Claims Rates and Syncope Admission Rates From 2008 to 2017 Data were fitted using fractional polynomial regression analysis. The number of eligible syncope emergency department visits for each state-year was used as frequency weight (proportionate to the size of each circle). States with fewer than 5 emergency department visits per year were excluded from the plots. A, Malpractice claims rate. B, Syncope admission rate.

A substantial variation in the malpractice claims rate across states, ranging from 0.27 claims per 100 000 people to 8.63 claims per 100 000 people, was observed during the study period (Figure 2A). Admission rates varied widely across states, with a decreasing pattern (Figure 2B).

**Figure 3.**
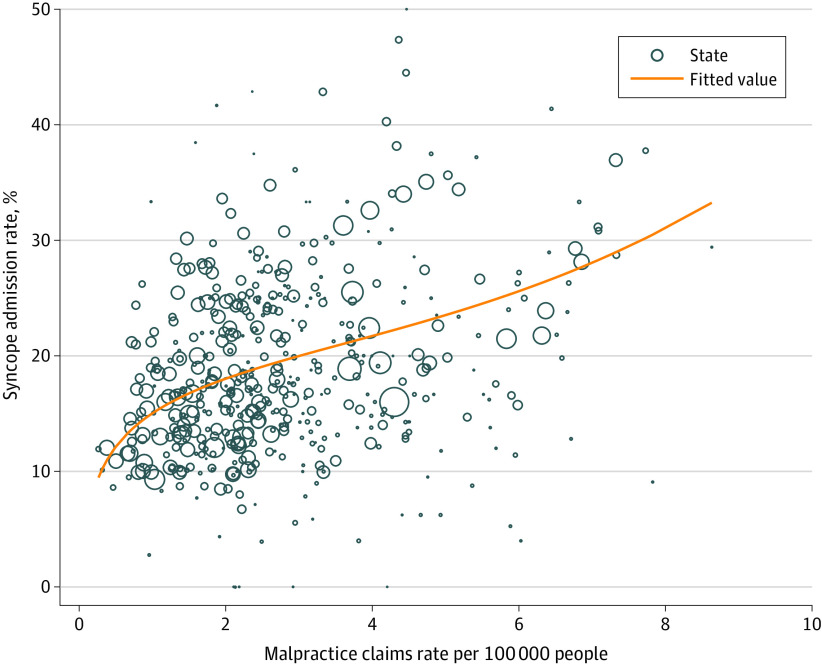
Association Between Malpractice Claims Rate and Syncope Admission Rate Measured by state-year. Data were fitted using fractional polynomial regression analysis. The number of eligible syncope emergency department visits for each state-year was used as frequency weight (proportionate to the size of each circle). States with fewer than 5 emergency department visits per year were excluded from the plot.

Results from the state-level fixed-effects model indicated that every 1 in 100 000–person increase in the malpractice claims rate was associated with an absolute increase of 6.70% (95% CI, 4.65%-8.75%) or a relative increase of 35.6% in the hospital admission rate (Table 2 and Figure 3), which represented $102 182 828 in hospital costs (calculated by multiplying 234 750 lower-risk syncope ED visits by the 6.70% change in admission rate, which was then multiplied by the $6542 in additional hospital costs associated with a hospital admission) associated with lower-risk syncope in the cohort. This same fixed-effects model indicated no association between the malpractice claims rate and the admission rate (coefficient, 0.09%; 95% CI, −6.61% to 6.79%) for the 2029 patients who had a first ED visit for appendicitis during the study period (Table 2).

**Table 2.** State-Level Fixed-Effects Regression Analysis of Malpractice Claims Rate and Admission Rate

Dependent variable	Admission rate, coefficient % (95% CI)	
	Lower-risk syncope	Appendicitis
Malpractice claims rate per 100 000 people	6.70 (4.65 to 8.75)^a^	0.09 (−6.61 to 6.79)
Proportion of female patients	−0.23 (−0.46 to −0.004)^b^	−0.15 (0.11 to −0.15)
Proportion of patients in age category, y		
18-64	−0.15 (−0.29 to −0.01)^b^	−0.13 (−0.33 to 0.08)
65-75	1 [Reference]	1 [Reference]
≥76	−0.23 (−0.36 to −0.10)^a^	−0.07 (−0.28 to 0.14)
Constant	27.43 (14.46 to 40.39)^a^	76.59 (56.84 to 96.34)^b^
Patients, weighted No.	233 127	2029

^a^ *P* < .001.

^b^ *P* *<* .05.

## Discussion

In this study cohort, we found that an increase in the malpractice claims rate was associated with a substantial increase in the hospital admission rate of low-risk patients with syncope. To our knowledge, this study is the first to report that physician behavior is associated with the liability risk climate. We found an increase of 6.7% in the hospital admission rate for every 1 in 100 000–person increase in the malpractice claims rate, which represented estimated extra costs of $102 million in this study population alone. Given a modest representation of private insurance among the Medicare population (mostly through Medicare Advantage), the potential impact among fee-for-service Medicare enrollees could be more significant than that reported in the present study. Using a lower-risk syncope cohort of patients who are generally discharged from the ED,^28,29^ our study’s findings indicate an association between malpractice claims rates or liability risks and unnecessary hospital admissions because of the practice of defensive medicine.

The hospitalization costs of the extra admissions in this cohort were substantial, but the true costs of defensive medicine may be higher.^30^ Defensive medicine, which may expose patients to unnecessary procedures and hospital admissions, incurs substantial nonmonetary and indirect costs in addition to the direct costs of extra tests and admissions estimated in this study.^14,31,32^ The costs of false-positive test results and hospital admissions associated with further testing and unnecessary treatments are well documented. They include the risks of hospitalization and possible subjection to medical errors and hospital-acquired infections.^33,34^ These costs are real and are associated with harms that are difficult to quantify and not reflected in the costs estimated in this study.

Consensus has been reached that there is little value in the hospitalization of low-risk patients with syncope.^28^ While a small number of low-risk patients with syncope may need to be admitted to the hospital for social or other reasons, most guidelines specifically recommend that patients with syncope who are at low risk of serious outcomes not be admitted.^28,29^ Furthermore, there is established evidence to guide clinicians in identifying low-risk patients who could be discharged.^35,36,37,38^ We assembled a lower-risk cohort and used standardized codes that have high specificity and good sensitivity for syncope,^22^ excluding any patient with potentially serious comorbid conditions. We do not suggest that our stratification was precise because unobserved factors may have been associated with admission decisions, but we are confident that these patients would likely have been candidates for discharge per existing guidelines or good clinical practice based on the admission rate of 18.8% for this lower-risk group.

The admission rate from the present cohort may appear high, but it reflects the reality of physician practice in the US. Previous research found that physicians have good judgment with regard to identifying patients with syncope who are likely to develop serious outcomes.^39^ Contrary to their judgment, physicians still admitted 28% of patients they deemed low risk in that cohort. No physician wants an adverse outcome, and there are many reasons physicians may be uncomfortable with discharging low-risk patients. Liability concerns may be only a part of this discomfort, and physicians in that study were not asked why they admitted low-risk patients. While the overall admission rate for the lower-risk cohort in the present study was high, a pattern of decreasing annual admission rates was observed over time (Figure 2B). This decreasing pattern seems to align with the publication and dissemination of clinical decision support and expert recommendations over the study period.^28,35,40,41,42^

It is fair to surmise that risk-averse behavior is likely associated with the admission of low-risk patients when guidelines and risk scores recommend that these patients not be admitted. Furthermore, this behavior likely has implications for the variability in syncope admission rates within the US and around the world, with the risk of malpractice litigation being especially high in the US.^9^ The admission rates for syncope (of varying risk levels) are reported to be 34% to 59% in California, 47% in Utah, 69% in Massachusetts, and 83% in New York.^35,40,43,44^ These rates compare internationally with admission rates of less than 30% in Australia and Italy and less than 10% in Canada.^36,42,45^ The malpractice climates are substantially different in these states and countries, as are the health care systems, which also likely play an important role in the decision to admit patients.^9,46^ Given the complexity, it would be difficult, if not impossible, to identify the sole factor associated with the malpractice climate that would account for the differences observed in syncope admission rates.

Most previous studies have used tort reform rather than malpractice claims rate as a proxy for the liability climate when studying physicians’ behavior regarding other conditions, and these studies have reported variable results.^10,11,12^ There are several drawbacks to using tort reform as a proxy for physicians’ perceptions of malpractice risk.^13^ First, there are several levels of tort reform. Some payment caps are greater than $1 to $2 million and may have little association with the pursuit of malpractice litigation. Second, with policy changes and the implementation of payment caps, the consequences of litigation for the liability climate and physicians’ behavior may take time to manifest. In addition, some states fluctuate in their positions on tort reform, preventing any possibility of real change in the malpractice climate. We believe that the actual malpractice claims rate is likely a better proxy for physician-perceived liability climate.^31^ Third, any risk of litigation, regardless of payment cap amount, may engender some level of fear in physicians.

### Limitations

Clinformatics Data Mart is a robust deidentified database of individuals with commercial or Medicare Advantage insurance coverage, so it does not include those who are uninsured or have Medicaid coverage. However, it is unclear to what extent such selection would have changed our estimation of the association between the malpractice claims rate and the syncope admission rate. We were also not able to consider practitioner factors unavailable in the data set (such as years of training and board certification) that may have been associated with physician risk and decision-making. However, we believe our findings are robust to any practice type (eg, academic, community, rural, and member-based systems) and physician factors (eg, experience and training), which are all represented in the national cohort. The National Practitioner Data Bank is also limited, as it does not include all payments (eg, settlements) and other burdens of the liability system, such as being included in a malpractice claim and having to defend oneself. However, we believe the claims activities in the National Practitioner Data Bank are a good measure of the state malpractice environment and a better proxy than state tort reform.

We assembled a lower-risk cohort retrospectively, without the availability of important variables, such as the prospective results of electrocardiographic evaluations, that are used in syncope risk assessment. We likely lacked the precision of prospective risk tools to identify low-risk patients. However, the purpose of this study was not to develop a cohort definition that physicians could use to identify low-risk patients. Our goal was to identify a cohort of patients with lower-risk syncope among whom most were discharged from the ED and for whom physician judgment would have been required for hospital admission decisions. This cohort allowed us to assess the potential role of the malpractice environment in decision-making when judgment was involved. Furthermore, we used appendicitis admissions as a negative control to indicate that the malpractice claims rate was not associated with decisions about when most patients should be admitted and that physician judgment would have had few implications for decision-making.

## Conclusions

In this study, admission rates for ED patients with low-risk syncope were associated with higher malpractice claims rates, which was likely associated with the practice of defensive medicine. Defensive medical decision-making is a learned behavior and may be impacted by the local claims rate and the liability risk climate. Although defensive medicine has likely existed in some form for years, the underlying factors and consequences have been elusive. Physicians’ risk-averse behaviors may be associated with many factors, but such behaviors come with substantial costs, as indicated by the findings of this study.
